# P11 - Immunological changes of frequently ill children with acute viral infections

**DOI:** 10.1186/2045-7022-4-S1-P66

**Published:** 2014-02-28

**Authors:** Amaliya Ayyubova, Maleyka Karimova, NA Magerramov

**Affiliations:** 1 Azerbaijan Medical University, Baku, Azerbaijan

## Background

Viral infections of the respiratory tract represent a major cause of morbidity in childhood.

**The aim** of our study was to identify the immunological changes in frequently ill children (FIC) with acute viral infections.

## Material and methods

To this end, were examined 340 FIC with respiratory diseases and 125 seldom ill children for comparison. Patients were examined in the acute period of the disease and in the period clinical remission. In a study of children number of CD3-cells, CD4-cells, CD8-cells, CD19-cells, the content of serum immunoglobulins A, M, G,E, the content of cytokines IL-1beta , TNF- alpha, IL-2, IL-6, IL-8, IFN-gamma, microbiocenosis upper respiratory tract and intestinal.

## Results

Our results show that in the acute period of the respiratory disease, reduced the level of cellular immunity (mainly, CD3-cells, CD4-cells and the index immunoregulator cells), marked imbalance of humoral immunity reduction of IgA and IgG, increase of IgM and IgE. By the marked in cytokine status increase proinflammatory cytokines IL-1beta, TNF-alpha, IL-6, IL-8 and reducing IL-2 and IFN-gamma. We found that the clinical remission of respiratory diseases in FIC is not accompanied by a normalization parameters of the immune system and cytokine status. High level of proinflammatory cytokines in the period of clinical remission, reflect ongoing inflammation, which is associated with persistence of the infection agent. Of these infections in our patients revealed chlamydia, mycoplasma, cytomegalovirus, staphylococcus aureus, candida albicans. Our FIC 67. 6% of cases in observed dysbiosis, the severity of which correlated decrease the immune system.

## Conclusion

Saved changes in the clinical remission in frequently ill children with acute viral infections requires adequate therapy.

